# The crossover from microscopy to genes in marine diversity: from species to assemblages in marine pelagic copepods

**DOI:** 10.1098/rstb.2019.0446

**Published:** 2020-11-02

**Authors:** Silke Laakmann, Leocadio Blanco-Bercial, Astrid Cornils

**Affiliations:** 1 Helmholtz Institute for Functional Marine Biodiversity at the University of Oldenburg (HIFMB), Ammerländer Heerstrasse 231, 26129 Oldenburg, Germany; 2 Alfred Wegener Institute Helmholtz Center for Polar and Marine Research, Am Handelshafen 12, 27570 Bremerhaven, Germany; 3 Bermuda Institute of Ocean Sciences, 17 Biological Station, GE 01 St George's, Bermuda

**Keywords:** identification, biodiversity, genetics, species, community, integrative approach

## Abstract

An accurate identification of species and communities is a prerequisite for analysing and recording biodiversity and community shifts. In the context of marine biodiversity conservation and management, this review outlines past, present and forward-looking perspectives on identifying and recording planktonic diversity by illustrating the transition from traditional species identification based on morphological diagnostic characters to full molecular genetic identification of marine assemblages. In this process, the article presents the methodological advancements by discussing progress and critical aspects of the crossover from traditional to novel and future molecular genetic identifications and it outlines the advantages of integrative approaches using the strengths of both morphological and molecular techniques to identify species and assemblages. We demonstrate this process of identifying and recording marine biodiversity on pelagic copepods as model taxon. Copepods are known for their high taxonomic and ecological diversity and comprise a huge variety of behaviours, forms and life histories, making them a highly interesting and well-studied group in terms of biodiversity and ecosystem functioning. Furthermore, their short life cycles and rapid responses to changing environments make them good indicators and core research components for ecosystem health and status in the light of environmental change.

This article is part of the theme issue ‘Integrative research perspectives on marine conservation’.

## 1. Introduction

### (a) Biodiversity and species identification

Biodiversity describes the variations that are found within communities, which includes variability within species, between species and between ecosystems and, as such, it is key to ecosystem functioning. Understanding biodiversity and its change constitutes the basis for conservation and management of marine biodiversity in times of perceptible changes in marine systems. For the marine pelagic realm, our current understanding of patterns in metazoan planktonic biodiversity results from decades of work by oceanographers, ecologists and taxonomists.

The identification and delimitation of species are based on various criteria that evolved from different species concepts [1]. Correct species identification is a prerequisite for most biological studies and has been traditionally based on morphological diagnostic characters. Extensive knowledge of the available reference literature and taxonomic experience are essential. To identify species independently of taxonomic expertise molecular methods have been used increasingly over the past decades. These analyses allow for a new perspective on plankton diversity and have called into question assumptions on biogeographic patterns and evolutionary relationships owing to the presence of cryptic or pseudo-cryptic species (sibling species with inconspicuous or non-existent morphological differences) within many taxa [2–4]. These observations imply that traditional species concepts based on morphological identified taxa may have greatly underestimated species richness [5]. Recent efforts have produced an enormous wealth of novel data from high-throughput metagenomic sampling on plankton distribution and diversity [6–9], and revealed that a large fraction of the recorded plankton diversity belongs to still unknown taxonomic groups [9]. These results illustrate that we still have too little knowledge and understanding of the diversity of plankton and their relationship to abiotic and biotic factors, especially in the light of environmental change.

To outline methodological trends in identifying, analysing and recording marine biodiversity, the present review provides an overview of the transition from morphological to molecular identification methods. For this, we use planktonic copepods as model taxon as they often dominate zooplankton communities and, as such, play a crucial role in marine systems.

### (b) Copepods as model taxon

Planktonic copepods are known for their high taxonomic and ecological diversity, making them one of the most studied marine taxonomic groups [10]. Studies comprise their biodiversity [11,12], morphology [13], taxonomy [14], phylogeny [15,16], phylogeography and distribution [17,18], life cycle strategies [19], feeding behaviour [20] or adaptation to various environmental conditions [21]. Copepods display a huge variety of behaviours, forms and life histories. They often dominate zooplankton communities, revealed by both morphological and molecular assessments, and constitute an important part in marine food webs. As such, they have an important role in the energy transfer in most marine ecosystems [22]. Owing to short life cycles and rapid responses to changing environments, they are good indicators for ecosystem health and status [23]. Therefore, the identification of copepod (and zooplankton) community composition and structure (i.e. identification of dominant species and diversity) is important to understand and to monitor changes in marine systems [23–26].

## 2. Morphological species identification and biodiversity assessments

Planktonic copepods are the most abundant metazoans on Earth [27]. In consequence, plankton ecologists who investigate the composition or diversity of copepod communities from plankton net samples often need to identify thousands of individuals in a delimited time period. Traditionally, morphological structures are the primary tool to identify copepod species. These are usually morphological characteristics of the exoskeleton and, owing to the small size of most copepod species, these characteristics are only visible microscopically. Owing to the high number of individuals, in routine identifications only a few diagnostic characters are used to identify species [28].

Since publication of the Systema Naturae by Linnaeus [29] in 1735 more than 14 000 copepod species have been described, including more than 2000 planktonic species [27,30]. During the ‘Golden Age’ of copepod taxonomy, between the late nineteenth century to the middle of the twentieth century, large volumes emerged that were dedicated to the description of copepod species from large expeditions, e.g. [31–36]. Until today, these volumes are the foundation for species identification in marine planktonic copepods, and often their drawings are just reproduced with no changes in modern treatises. Further important volumes with species descriptions have been published in the later twentieth century, e.g. [37–42]. Identification keys exist either for copepod species of certain regions (e.g. [43,44]) or for single families or genera [42,45–48]. In 2004, a first comprehensive overview of all copepod families (also non-planktonic) was published [14] providing identification keys for genera of each family with standardized drawings. As the discovery of new species is continuing, affiliations of already described species may be subject to taxonomic revisions because when new related species are discovered, this often requires a redefinition of the taxonomic characters for the whole group, with the associated outdating of the existing keys. In consequence, the standard references are often outdated for many accepted species names and the preciseness of identification is highly dependent on the taxonomic expertise of the analyst. Species descriptions within a family or genus are often incoherent as many different authors have described the species, which complicates the evaluation of whether the specimens under consideration belong to a known species or are new to science. Furthermore, species descriptions are generally based on morphological characters of adult specimens and are also gender-related, which makes it nearly impossible to identify early (nauplii) and juvenile (copepodite) life stages, both more abundant than adults. Lists of currently accepted species are compiled and updated at the *World Register of Marine Species* (WoRMS, [29]) and the *Marine Planktonic Copepods* webpage [49], with the latter also including drawings and biogeographic notes for each species.

A limitation on the species discrimination and definition based on morphological characters is the subjective nature of diagnostic characters. Unless crossing experiments are carried out, the definition of a species' limits, identity and associated diagnostic characters are always subject to the criteria of the taxonomist. That is, whatever characters are selected as those that draw the line between species rather than mere variability or morphotypes within a species depends not only on the data available to the researcher, but also on the researcher's subjective opinion of what a species is. Furthermore, the discrimination of sibling species is often not even possible (e.g. within the prominent genus *Calanus*), at least using characters that could be used on a routine basis without resourcing to complicated microscopy procedures such as the study of tegumental pores [50,51]. Thus, rare species may be overlooked or co-occurring sister species that differ only in minuscule characteristics may be merged.

The sampling method also has a great impact on the preciseness of identification. Often swimming and mouth appendages hold the features that characterize species in copepods. In net samples, these appendages are often broken and may thus only allow identification of the specimen to the genus level. Large mesh sizes (greater than 200 µm) have also led to more intensive studies on larger calanoid copepods than on the often more abundant cyclopoid copepods [52,53]. Recent geometric morphometric approaches allow the avoidance of problems arising from missing morphological characters [54], but are difficult to implement in routine identifications. For all these reasons, juveniles and non-calanoid copepods are often grouped to higher taxonomic levels [55], which results in underestimating species diversity and richness. To facilitate routine identification, techniques based on machine learning have been developed to semi-automatically identify and quantify the composition of plankton assemblages from images of preserved samples at a relatively coarse taxonomic level (ZooScan [56], EcoTaxa [57]). These techniques extract not only useful information on abundance, but also several metrics that allow the estimation of individual sizes.

In summary, morphological species identification of copepod samples not only enable the study of taxonomic diversity but also provide information on abundances, biomass, size class distribution and life stage composition (table 1). Next to this, the study of organisms allows the collection of data on species traits and thus functions in marine systems. Species identification is, however, hampered by the condition of the organisms and the level of taxonomic expertise. If several analysts with a different experience level identify species from sets of samples, e.g. in long-term monitoring efforts, the list of species and stages counted and identified vary with taxonomic changes and increasing expertise, resulting in many different taxonomic entities [58].

**Table 1.** Potentials and drawbacks for the traditional morphological and molecular genetic species identification in biodiversity analyses.

	potentials	drawbacks
morphological identification		
	# information on — life stage composition and size class distribution — traits and hence ecological role/function — quantification (abundance, biomass)	# requires taxonomic expertise on diverse groups # incoherent species descriptions # gender- and stage-related diagnostic species characters # identification depends on condition of the organism # subjective nature of diagnostic characters # no identification of sibling and cryptic species and populations # time intensive
molecular genetic identification		
single species	# species identification of young developmental stages and cryptic/sibling species → higher diversity # identification of populations # standardized identification, automation	# requires prior methodological knowledge
metabarcoding	# simultaneous identification of a multitude of species # processing of large numbers of samples # efficient and cost-effective for analysing bulk samples # standardized identification, automation	# no information on — community structure regarding size and stage distribution — biomass and abundance — ecological role # depends on high-quality sequence reference database for different regions and progress in providing sequence reference entries # identification of thresholds # false positives, false negatives # primer and PCR biases (amplicon sequencing)

## 3. Molecular techniques to address diversity

### (a) Molecular identification of single species

To help in addressing one or several of the previous questions and challenges using morphological identification of species, and starting by the end of the past century, several molecular methodologies have been developed for the study of Copepoda. The first methods used for species discrimination were based in variations of fragment length analyses. Most of the methods were developed in the 1980s in the biomedical field [59,60] and successfully applied to other fields soon after their discovery, including many applications to the species or lineages discrimination. For copepods, some of the first groups to benefit from these methods were the ecologically relevant *Pseudocalanus* [61,62] and the North Atlantic *Calanus* species complexes [62–64] and later on other species complexes at local or regional level [65,66]. Two main methods were used for these pioneering studies. Meanwhile, a species-specific PCR, based on competitive priming between species-specific primers [60], was the method used in some studies [60–62,64] and a restriction fragment length polymorphism (RFLP; [59]) was the approach developed by others [63]. All studies were developed on mitochondrial genes (16S rRNA, cytochrome c oxidase subunit I (COI)) to take advantage of the existing high between-species variability and relatively conserved within-species variability. These techniques are robust, time-efficient and of low costs (both in terms of daily expenses (consumables) and equipment required, with just a thermocycler and a gel system needed). On the other hand, to develop a reliable method, an in-depth knowledge of the genetic diversity of the studied species is needed, to ensure that the regions targeted by the restriction enzyme or those binding to the species-specific oligonucleotides are highly conserved within the species. In marine copepods, with population sizes often in the range of billions to trillions, it would be very difficult to ensure that all individuals would show a conserved region long enough to fit the enzyme site or the oligonucleotide region [66] and coverage should include all known populations to consider private alleles. Furthermore, they are not easily expandable, and the addition of any new species would usually involve having to develop the protocol from scratch. Another problem associated with these methods is the lack of ability to detect cryptic species, since they can be mistaken for one of the existing species (if the target region(s) is/are identical) or for a negative result (if none of the regions are conserved). Despite the rise of DNA-sequence based methods soon after (which overcome some of the aforementioned problems), still some related methods have been developed in recent years. Amplicon length variability, based on insertion/deletion markers, has been used to discriminate between all North Atlantic *Calanus* species [67,68]. This method comprises a number of different indel regions, and is robust against the failure of one of the markers in case of priming site variability, since the remaining markers would allow species assignment. With the advantage of little time and budget investment required, these methods were used, for example, to characterize the distribution of the different *Calanus* species in the North Atlantic [68,69].

Similarly, the analysis of DNA fragment lengths such as multilocus microsatellite fingerprinting was also used to discriminate between sister species [70] and in combination with DNA sequencing of mitochondrial genes, to support the presence of cryptic speciation within a taxonomically complex species [71]. This method gives insights between sister species in a greater genetic resolution and allows parallel studies on gene flow and dispersal. However, the major drawback of this approach is the intensive development of microsatellite markers specific for every species, and the need to re-develop the method from scratch when adding and combining several species to avoid ascertainment bias. More recently, the use of genome-wide single nucleotide polymorphisms (SNPs) has been opening a new door to discriminating between cryptic species clusters with high genetic diversity and sympatric mitochondrial DNA clades [72].

With the refinement of DNA sequencing, which is known nowadays as DNA barcoding, the species identification based on the sequence of a relatively short fragment of DNA [73,74] was developed in the early 2000s. This method was already used previously to differentiate between species or forms of marine copepods [2,75–77]. Although different authors made use of a number of different markers, especially the mitochondrial genes (16S, COI), with the launch of the Consortium for the Barcode of Life initiative [74], the use of the Folmer region [78] of the COI was the chosen for metazoan (copepods included) barcoding. The original objective of DNA barcoding was not to address taxonomic questions (DNA taxonomy) but just developed as an identification tool (see review by [79]). Within this scope, several initiatives were oriented to provide a database of known species, providing global or regional molecular references based on morphologically identified individuals by taxonomic experts, often flagging some potential cryptic speciation issues [12,80,81], a hidden diversity that could not be addressed by morphological methods (table 1).

But, even before the launch of the barcoding initiatives, the use of sequences was often oriented as a tool to aid in solving taxonomic problems—ideally as a complement to morphological studies [82]—to understand the cryptic diversity within Copepoda, which is indiscernible by morphological characters. Compared to fragment length-based methods, the use of DNA sequences (independently of the marker) allowed scientists to study other facets of the biology and the taxonomy of species, such as degree of relatedness between species (by distance methods or phylogenetic reconstructions), to detect, reject or support the presence of cryptic species, and even to delineate genetically isolated subpopulations within a species. Many of the previously mentioned molecular studies require detailed morphological information and the combination of both is nowadays known as integrated taxonomy. Within this approach, information on both the morphological and molecular species identification is paired at individual level, and ideally stored in open-source sequence reference libraries, such as BOLD (http://www.boldsystems.org/) or, (even better), paired with a museum collection. Such an approach has been very fruitful for characterizing cryptic diversity of open ocean copepods [2,4,83–85], a diverse range of species complexes [3,86,87] especially when they are used as important indicators of climate responses [68,88], meso- and bathypelagic hidden diversity [89], the identity of key players in upwelling ecosystems [90–92], and for dealing with the always extreme complexity of non-calanoid copepods [18,93], for which the relevance in the ocean ecosystems has been always been understudied owing to the complexity of their taxonomy [53]. Meanwhile, while molecular methods alone are useful to detect isolated evolutionary lineages [12,94], without an accompanying taxonomic and morphological study it would be very difficult to use this information further to infer the ecological relevance of such hidden diversity, especially in the context of past studies.

### (b) Molecular identification of assemblages and communities

With the advent of high-throughput sequencing techniques, the molecular genetic identification of single metazoan species moved fast forward towards the analysis of whole marine metazoan communities such as meiofauna [95] or zooplankton [6,96]. Multiple species and entire communities, can be identified simultaneously by analysing orthologous gene regions in parallel from environmental samples using next-generation sequencing platforms. This process is defined as metabarcoding. Compared to DNA barcoding on single specimens, metabarcoding is based on shorter gene fragments, and in general of a single marker, often variable regions of conserved nuclear small-subunit ribosomal RNA genes 18S rRNA (V1-2 [6,96,97]; V4 [98,99]; V9 [9,100–107] and 28S rRNA [7,8]). Owing to their conserved nature, resolving the obtained sequences to identify species is often impossible, since the same sequence for that region might be shared between genera, families or even superfamilies, depending on the marker used and the phylogenetic divergence between the different species. Mitochondrial markers allow a better taxonomic resolution and species identification compared to nuclear markers [108–112]. However, owing to the less conserved primer regions in COI it also implies primer mismatches and consequently missing amplification of a wide range of taxa (false negatives). Possible solutions to this problem are multi-marker and multi-primer approaches [111,113] as they enable the identification to different taxonomic levels and of a greater proportion of different taxa and thus biodiversity.

Successful species assignment also requires a complete and high-quality reference sequence database, ideally for the location and the season. For copepods, there are a limited number of high-quality DNA barcodes available for the identification [12,79,114] and such a shortage may lead either to misidentification or to an underestimation of diversity owing to non-identification of the sequences obtained [100,110]. When DNA reference sequences do not exist and sequences cannot be identified, similarity/divergence thresholds are chosen to cluster sequences into taxonomic units [95,98,110]. The choice of the threshold depends on the divergence in the chosen gene fragment between species, genera and families in closely- and distantly-related organisms, and has an influence on the number or taxa detected by metabarcoding.

**Figure 1.**
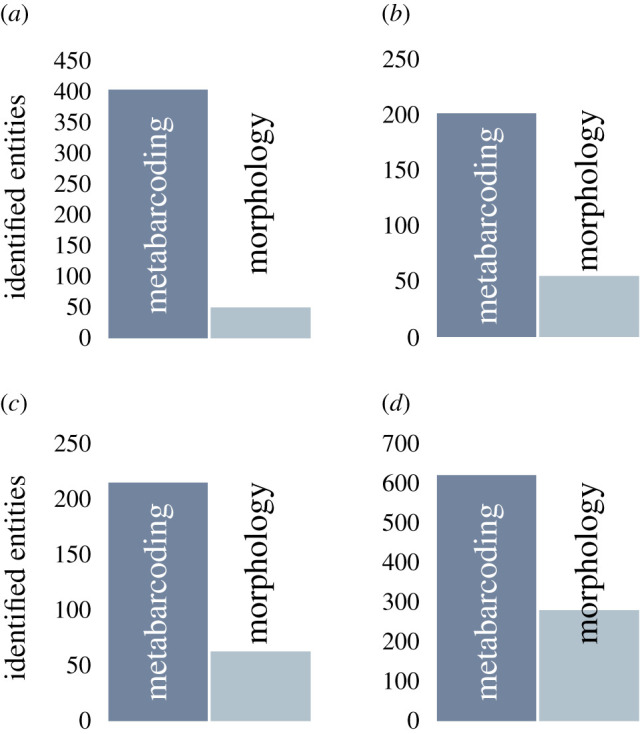
Higher diversities from bulk zooplankton samples using the metabarcoding approach (number of operative taxonomic units, OTUs) compared to morphological identification (identified to different taxonomic levels). (*a*) 18S V9 metabarcoding and morphological identification to family, order or phylum [109], (*b*) 18S V1–2 metabarcoding (97% similarity threshold) and morphological identification generally to species or genus level and in particular meroplanktonic larvae to major taxonomic groups [6], (*c*) 18S V7–V9 metabarcoding (97% similarity threshold) and morphological identification to species level or lowest-ranking taxon possible [115], (*d*) 18S V9 metabarcoding (97% similarity threshold) and morphological identification to genus level where possible [105].

Despite these discussed challenges, metabarcoding provides a new and more comprehensive view on zooplankton and copepod biodiversity and assemblages by detecting hidden diversity and by offering the possibility of automation and a cost-effective analysis to be applied in time-series analysis and ocean ecosystem assessments. Metabarcoding analyses have been a major breakthrough in identifying bulk samples of zooplankton and thus copepod assemblages as they detect many different species from diverse taxa, cryptic species or developmental stages (e.g. meroplankton, nauplii, copepodites), previously hidden by morphological identification only (see §2), resulting in higher number of taxa or diversities (figure 1) [6,7,96,105,107,110,115]. Moreover, rare and non-indigenous species, among others, are more likely to be identified by a molecular genetic approach [103]. Several studies on zooplankton have also demonstrated the ability of metabarcoding to map both temporal and spatial patterns in zooplankton diversity and assemblages [8,100,106,107,110,111].

A major drawback of metabarcoding of bulk samples compared to morphological analysis is that quantitative ecological parameters that characterize communities such as the composition of life stages, abundances and biomass cannot currently be assessed. For such a quantitative analysis, the correlation between the number of sequence reads and the biomass or the abundance of the individual species is still in discussion, since biases might exist owing to, for example, the differences in gene copy numbers, or to PCR bias, in which the chosen primer may match better in some taxa and thereby leads to a better amplification of these specific taxa (primer match/mismatch). Despite these caveats, analysing mock assemblages of pelagic copepods (on family level) showed a significant correlation of the number of sequence reads to the dry weight of the taxon [7]. In real samples, sequence numbers and counts of calanoid copepods showed a significantly positive correlation [107] and a high correspondence in whole zooplankton samples [103]. Since the number of reads would only reflect relative abundances (there is no relationship between amount of DNA extracted and number of reads obtained after sequencing) some other analyses (DNA quantification by quantitative PCR, addition of internal DNA standards at DNA extraction or amplification, identification of mock communities, biomass measures, among others) would still be needed to move from read number to a biomass-like measurement.

Next to genetic material from whole zooplankton samples, multiple species can also be identified from genetic material sampled from the environment, referred to as environmental DNA (eDNA). By definition, eDNA is the DNA extracted from an environmental sample such as water, soil or air without isolating the target organism [116]. Large-scale biodiversity surveys based on eDNA and using both nuclear ribosomal and/or mitochondrial markers have shown that this methodology can provide valuable insights in the zooplankton and copepod communities, including potential invaders [117,118].

A comparative approach on zooplankton, including morphological identification and multi-marker metabarcoding of bulk samples and eDNA, revealed significant differences in taxonomic compositions. However, the dominant copepod taxa (identified to the family level) were identified in all of the three different approaches [109]. Metabarcoding of bulk samples gave a better measure of the zooplankton (and the morphological identification) itself, but eDNA metabarcoding better reflected the overall diversity of the broader marine community, which is not as accessible and easy to sample as zooplankton. To improve the detection of organisms in metabarcoding of eDNA, best-practices, ranging from field to laboratory and data processing standards, are still needed. These would include minimum reporting standards regarding study design, water collection, sample preservation, extraction process and high-throughput sequencing [119]. Furthermore, raw data (FASTQ files) and processing data pipelines should be made available for the scientific community by the storage in complementary repositories to allow full transparency and reproducibility (outlined by [120]).

## 4. Conclusion

Our understanding of pelagic biodiversity results from decades of morphological taxonomic work; however, this knowledge has to be integrated into novel molecular genetic approaches. Only by adjusting and linking these new tools with the traditional methods can we maintain the acquired knowledge for future research using molecular data as the main workhorse for community ecology and taxonomy. The power of a so-called total evidence approach by identifying plankton, relying on both molecular and morphological information whenever possible (also referred to as ‘successful marriage of molecular and morphological methods') was already outlined one decade ago [28]. At that time, the authors outlined: *It has also been suggested that advances in sequencing technologies may overtake the single-gene barcode approach by enabling rapid genomics of species, even during routine sampling, making the current mitochondrial-based barcoding seem not ambitious enough* [28, p. 1121]. Nowadays, in the times of high-throughput sequencing techniques, we are able to analyse whole communities based on molecular data, but it is still advisable to integrate morphological approaches to ensure identification, and especially quantification. For the simultaneous identification of multiple species in whole samples, the comparison between morphological and molecular identification by metabarcoding confirms that the molecular approach is not yet ready to completely replace traditional taxonomy by morphological analyses [110]. Hence, metabarcoding still needs the ground truthing by the direct comparison to the traditional morphological taxonomic analysis of the sample [107], especially for quantification [121].

For zooplankton, molecular genetic studies demonstrate promising diversity analyses based on bulk samples and allow the processing of large numbers of samples, which is advisable (in combination with traditional methods, table 2) for future studies on planktonic communities. However, three checkpoints should be implemented in future metabarcoding studies on plankton: protocol optimization, error minimization and a downstream analysis that considers potential and remaining biases [121]. To analyse marine life across groups, communities, taxa or environments that are not as accessible and easy to sample as zooplankton, eDNA analyses constitute a promising, non-invasive and non-destructive methodology to provide insights into marine life, especially when the organisms are rare, big, elusive, threatened, endangered, non-indigenous or cryptic. Particularly for metabarcoding of eDNA to detect macroorganisms, we need best-practices ranging from field to laboratory and data processing standards [121].

**Table 2.** Guidelines to select approach to specific research questions.

research question	approach
cladistics and systematics	integrative analyses
ecological studies on community structure	morphological identification metabarcoding integrated approach
single/few sister species distribution	morphological identification, fragment analysis
alpha-diversity	metabarcoding
monitoring	metabarcoding morphological identification (for community structure) integrative approach
invasive species	metabarcoding (bulk samples and eDNA)

Looking back on the fast development and improvement in the field of species, assemblage and community identification, the continuous methodological advancement of sequencing technologies and bioinformatics will, if validated by traditional methods, allow a more comprehensive view on marine life to be included in marine conservation and management.

## Data accessibility

This article has no additional data.

## References

[1] De Queiroz K 2007 Species concepts and species delimitation. Syst. Biol. 56, 879–886. (10.1080/10635150701701083) 18027281

[2] Goetze E 2003 Cryptic speciation on the high seas; global phylogenetics of the copepod family Eucalanidae. Proc. R. Soc. B 270, 2321–2331. (10.1098/rspb.2003.2505) PMC1691510 14667347

[3] Cornils A, Held C 2014 Evidence of cryptic and pseudocryptic speciation in the *Paracalanus parvus* species complex (Crustacea, Copepoda, Calanoida). Front. Zool. 11, 19 (10.1186/1742-9994-11-19) 24581044 PMC3948017

[4] Bode M, Laakmann S, Kaiser P, Hagen W, Auel H, Cornils A 2017 Unravelling diversity of deep-sea copepods using integrated morphological and molecular techniques. J. Plankton Res. 39, 600–617. (10.1093/plankt/fbx031)

[5] Norris RD 2000 Pelagic species diversity, biogeography, and evolution. Paleobiology 26 (4, Suppl.), 236–258. (10.1666/0094-8373(2000)26[236:PSDBAE]2.0.CO;2)

[6] Lindeque PK, Parry HE, Harmer RA, Somerfield PJ, Atkinson A 2013 Next generation sequencing reveals the hidden diversity of zooplankton assemblages. PLoS ONE 8, e81327 (10.1371/journal.pone.0081327) 24244737 PMC3820580

[7] Hirai J, Kuriyama M, Ichikawa T, Hidaka K, Tsuda A 2015 A metagenetic approach for revealing community structure of marine planktonic copepods. Mol. Ecol. Res. 15, 68–80. (10.1111/1755-0998.12294) 24943089

[8] Hirai J, Tsuda A 2015 Metagenetic community analysis of epipelagic planktonic copepods in the tropical and subtropical Pacific. Mar. Ecol. Prog. Ser. 534, 65–78. (10.3354/meps11404)

[9] de Vargas C et al. 2015 Eukaryotic plankton diversity in the sunlit ocean. Science 348, 1261605 (10.1126/science.1261605) 25999516

[10] Mauchline J 1998 Advances in marine biology (eds Blaxter JHS, Southward AJ, Tyler PA). San Diego London Boston New York Sydney Tokyo Toronto: Academic Press.

[11] Beaugrand G, Ibañez F, Lindley JA, Reid PC 2002 Diversity of calanoid copepods in the North Atlantic and adjacent seas: species associations and biogeography. Mar. Ecol. Prog. Ser. 232, 179–195. (10.3354/meps232179)

[12] Blanco-Bercial L, Cornils A, Copley N, Bucklin A 2014 DNA Barcoding of marine copepods: assessment of analytical approaches to species identification. PLOS Curr. Tree Life 1, ecurrents.tol.cdf8b74881f87e3b01d56b43791626d2. (10.1371/currents.tol.cdf8b74881f87e3b01d56b43791626d2) PMC4073882 24987576

[13] Bradford-Grieve JM, Boxshall GA 2019 Partial re-assessment of the family structure of the Clausocalanoidea (Copepoda: Calanoida) using morphological data. Zool. J. Linnean Soc. 185, 958–983. (10.1093/zoolinnean/zly086)

[14] Boxshall GA, Halsey SH 2004 An introduction to copepod diversity. London, UK: Ray Society.

[15] Blanco-Bercial L, Bradford-Grieve J, Bucklin A 2011 Molecular phylogeny of the Calanoida (Crustacea: Copepoda). Mol. Phylogenet. Evol. 59, 103–113. (10.1016/j.ympev.2011.01.008) 21281724

[16] Laakmann S, Markhaseva EL, Renz J 2019 Do molecular phylogenies unravel the relationships among the evolutionary young “Brafordian” families (Copepoda; Calanoida)? Mol. Phylogenet. Evol. 130, 330–345. (10.1016/j.ympev.2018.10.028) 30366087

[17] Blanco-Bercial L, Álvarez-Marqués F, Bucklin A 2011 Comparative phylogeography and connectivity of sibling species of the marine copepod *Clausocalanus* (Calanoida). J. Exp. Mar. Biol. Ecol. 404, 108–115. (10.1016/j.jembe.2011.05.011)

[18] Cornils A, Wend-Heckmann B, Held C 2017 Global phylogeography of *Oithona similis* s.l. (Crustacea, Copepoda, Oithonidae) – a cosmopolitan plankton species or a complex of cryptic lineages? Mol. Phylogenet. Evol. 107, 473–485. (10.1016/j.ympev.2016.12.019) 28007567

[19] Ikeda T, Hirakawa K 1996 Early development and estimated life cycle of the mesopelagic copepod *Paraeuchaeta elongata* in the southern Japan Sea. Mar. Biol. 126, 261–270. (10.1007/BF00347451)

[20] Saiz E, Calbet A 2011 Copepod feeding in the ocean: scaling patterns, composition of their diet and the bias of estimates due to microzooplankton grazing during incubations. Hydrobiologia 666, 181–196. (10.1007/s10750-010-0421-6)

[21] Lee CE, Petersen CH 2003 Effects of developmental acclimation on adult salinity tolerance in the freshwater-invading copepod *Eurytemora affinis*. Physiol. Biochem. Zool. 76, 296–301. (10.1086/375433) 12905115

[22] Steinberg DK, Landry MR 2017 Zooplankton and the ocean carbon cycle. Annu. Rev. Mar. Sci. 9, 413–444. (10.1146/annurev-marine-010814-015924) 27814033

[23] Beaugrand G, Reid PC, Ibañez F, Lindley JA, Edwards M 2002 Reorganization of North Atlantic marine copepod biodiversity and climate. Science 296, 1692–1694. (10.1126/science.1071329) 12040196

[24] Richardson A 2008 In hot water: zooplankton and climate change. ICES J. Mar. Sci. 65, 279–295. (10.1093/icesjms/fsn028)

[25] Defriez EJ, Sheppard LW, Reid PC, Reuman DC 2016 Climate change-related regime shifts have altered spatial synchrony of plankton dynamics in the North Sea. Glob. Change Biol. 22, 2069–2080. (10.1111/gcb.13229) 26810148

[26] Brun P, Stamieszkin K, Visser AW, Licandro P, Payne MR, Kiorboe T 2019 Climate change has altered zooplankton-fuelled carbon export in the North Atlantic. Nat. Ecol. Evol. 3, 416–423. (10.1038/s41559-018-0780-3) 30742109

[27] Humes AG 1994 How many copepods? Hydrobiologia 292–293, 1–7. (10.1007/BF00229916)

[28] McManus GB, Katz LA 2009 Molecular and morphological methods for identifying plankton: what makes a successful marriage? J. Plankton Res. 31, 1119–1129. (10.1093/plankt/fbp061)

[29] Linnaeus C 1735 Systema naturae, sive Regna tria naturae. Systematice proposita per Classes, Ordines, Genera, & Species. Leiden, The Netherlands: Lugduni Batavorum.

[30] Walter TC, Boxshall GA 2020 WoRMS Copepoda: world of copepods database (v. 2019-03-05). In Species 2000 & ITIS Catalogue of Life, 2020-09-01 Beta (eds Y Roskov, G Ower, T Orrell, D Nicolson, N Bailly, PM Kirk, T Bourgoin, RE DeWalt, W Decock, E van Nieukerken, L. Penev). Digital resource at www.catalogueoflife.org/col. Leiden, The Netherlands: Species 2000.

[31] Claus C 1863 Die frei lebenden Copepoden mit besonderer Berücksichtigung der Fauna Deutschlands, der Nordsee und des Mittelmeeres. Leipzig, Germany: Verlag von Wilhelm Engelmann.

[32] Brady GS 1883 Report on the Copepoda collected by H.M.S. Challenger during the years 1873-76. *Report on the Scientific Results of the Voyage of HMS Challenger during the Years 1873-76, Zoology*. **8** (part 23), 1–142, pl. 1–55.

[33] Giesbrecht W 1892 *Systematik und Faunistik der pelagischen Copepoden des Golfes von Neapel und der angrenzenden Meeres-Abschnitte*. Berlin, Germany: Verlag Von R. Friedländer & Sohn.

[34] Sars GO 1901 Copepoda Calanoida, Parts I & II. Calanidae, Eucalanidae, Paracalanidae, Pseudocalanidae, Aetideidae (part). An account of the Crustacea of Norway, with short descriptions and figures of all the species. Bergen Museum 4, 1–26.

[35] Wolfenden RN 1911 Die marinen Copepoden der Deutschen Südpolar Expedition 1901–1903. II. Die pelagischen Copepoden der Westwinddrift und des südlichen Eismeers mit Beschreibung mehrerer neuer Arten aus dem Atlantischen Ozean. *Deutsche Südpolar-Expedition* (*Zoologie 4*) **12**, 181–380, plates 22–41, figs 1–82.

[36] Farran GP 1936 Copepoda great barrier reef expedition, 1928–1929. Sci. Rep. Br. Mus. (Nat. Hist.) 5, 73–142.

[37] Brodsky KA 1950 Copepoda Calanoida of the far eastern seas of the USSR and the Polar Basin. In *Identification keys to the fauna of the USSR**35* (ed. EN Pavlovsky), pp. 1–440. Moscow-Leningrad, Russia: Akademia Nauk USSR [in Russian].

[38] Tanaka O 1956 The pelagic copepods of the Izu region, Middle Japan. Systematic account I. *Families Calanidae and Eucalanidae*. Publications of the Seto Marine Biological Laboratory 5, 251–272. (10.5134/174553)

[39] Bradford JM, Haakonssen L, Jillett JB 1983 The marine fauna of New Zealand: pelagic calanoid copepods: families Euchaetidae, Phaennidae, Scolecithricidae, Diaixidae, and Tharybidae. Memoirs New Zealand Oceanogr. Inst. 90, 1–150.

[40] Bradford-Grieve JM 1999 The Marine Fauna of New Zealand: Pelagic Calanoid Copepoda. NIWA Biodiversity Memoir 111, 1–268.

[41] Nishida S 1985 Pelagic copepods from Kabira Bay, Ishigaki Island, southwestern Japan, with the description of a new species of the genus *Pseudodiaptomus*. Publ. Seto Mar. Biol. Lab. 30, 125–144. (10.5134/176098)

[42] Park T 1994 Taxonomy and distribution of the marine calanoid copepod family Euchaetidae. Bulletin of the Scripps Institution of Oceanography, University of California 29, 1–203, figs 1–91.

[43] Rose M 1933 Copépodes pélagiques. *Faune de France, Paris* **26**, 1–374, figs 1-456.

[44] Bradford-Grieve JM, Markhaseva E, Rocha CEF, Abiahy BB. 1999 Copepoda. In South Atlantic zooplankton, vol. 2 (ed. Boltovskoy D), pp. 869–1098. Leiden, The Netherlands: Backhuys Publishers.

[45] Frost BW, Fleminger A 1968 A revision of the genus *Clausocalanus* (Copepoda: Calanoida) with remarks on distributional patterns in diagnostic characters. Bull. Scripps Instn Oceanogr. tech. ser. 12, 1–235.

[46] Damkaer DM 1975 Calanoid copepods of the genera Spinocalanus and Mimocalanus from the central Arctic Ocean, with a review of the Spinocalanidae. Seattle, WA: U.S. Dept. of Commerce, National Oceanic and Atmospheric Administration, National Marine Fisheries Service.

[47] Park TS 2001 Taxonomy and distribution of the calanoid copepod family Heterorhabdidae. San Diego, CA: University of California Press.

[48] Böttger-Schnack R, Schnack D 2019 OncIdent—an interactive identification key for Oncaeidae Giesbrecht, 1893 [‘1892’] (Copepoda: Cyclopoida). Mar. Biodivers. 49, 1043–1046. (10.1007/s12526-018-0863-z)

[49] Razouls C, de Bovée F, Kouwenberg J, Desreumaux N. 2005--2019 Diversity and geographic distribution of marine planktonic copepods. Sorbonne University, CNRS. http://copepodes.obs-banyuls.fr/en [Cited 28 September 2019.].

[50] Fleminger A, Hulsemann K 1977 Geographical range and taxonomic divergence in North Atlantic *Calanus* (*C. helgolandicus*, *C. finmarchicus* and *C. glacialis*). Mar. Biol. 40, 233–248. (10.1007/BF00390879)

[51] Park JS, Mauchline J 1994 Evaluation of integumental pore signatures of species of calanoid copepods (Crustacea) for interpreting inter-species relationships. Mar. Biol. 120, 107–114.

[52] Paffenhöfer GA, Mazzocchi MG 2003 Vertical distribution of subtropical epiplanktonic copepods. J. Plankton Res. 25, 1139–1156. (10.1093/plankt/25.9.1139)

[53] Gallienne CP, Robins DB 2001 Is *Oithona* the most important copepod in the world's ocean? J. Plankton Res. 23, 1421–1432. (10.1093/plankt/23.12.1421)

[54] Karanovic T, Lee S, Lee W 2018 Instant taxonomy: choosing adequate characters for species delimitation and description through congruence between molecular data and quantitative shape analysis. Invert. Systematics 32, 551–580. (10.1071/IS17002)

[55] Cornils A, Schulz J, Schmitt P, Lanuru M, Richter C, Schnack-Schiel SB 2010 Mesozooplankton distribution in the Spermonde Archipelago (Indonesia, Sulawesi) with special reference to the Calanoida (Copepoda). Deep-Sea Res. Part II: Top. Stud. Oceanogr. 57, 2076–2088. (10.1016/j.dsr2.2010.09.011)

[56] Gorsky G et al. 2010 Digital zooplankton image analysis using the ZooScan integrated system. J. Plankton Res. 32, 285–303. (10.1093/plankt/fbp124)

[57] Picheral M, Colin S, Irisson J-O 2017 EcoTaxa, a tool for the taxonomic classification of images. http://ecotaxa.obs-vlfr.fr.

[58] Greve W, Reiners F, Nast J, Hoffmann S 2004 Helgoland Roads meso- and macrozooplankton time-series 1974 to 2004: lessons from 30 years of single spot, high frequency sampling at the only off-shore island of the North Sea. Helgoland Mar. Res. 58, 274–288. (10.1007/s10152-004-0191-5)

[59] Botstein D, White RL, Skolnick M, Davis RW 1980 Construction of a genetic linkage map in man using restriction fragment length polymorphisms. Am. J. Hum. Genet. 32, 314–331. 6247908 PMC1686077

[60] Gibbs RA, Nguyen PN, Caskey CT 1989 Detection of single DNA base differences by competitive oligonucleotide priming. Nucleic Acids Res. 17, 2437–2448. (10.1093/nar/17.7.2437) 2717399 PMC317634

[61] Bucklin A, Bentley AM, Franzen SP 1998 Distribution and relative abundance of *Pseudocalanus moultoni* and *P. newmani* (Copepoda: Calanoida) on Georges Bank using molecular identification of sibling species. Mar. Biol. 132, 97–106. (10.1007/s002270050375)

[62] Bucklin A, Guarnieri M, Hill RS, Bentley AM, Kaartvedt S 1999 Taxonomic and systematic assessment of planktonic copepods using mitochondrial COI sequence variation and competitive, species-specific PCR. Hydrobiologia 401, 239–254. (10.1023/A:1003790411424)

[63] Lindeque PK, Harris RP, Jones MB, Smerdon GR 1999 Simple molecular method to distinguish the identity of *Calanus* species (Copepoda: Calanoida) at any developmental stage. Mar. Biol. 133, 91–96. (10.1007/s002270050446)

[64] Hill RS, Allen LD, Bucklin A 2001 Multiplexed species-specific PCR protocol to discriminate four N. Atlantic *Calanus* species, with an mtCOI gene tree for ten *Calanus* species. Mar. Biol. 139, 279–287. (10.1007/s002270100548)

[65] Kempter J, Piasecki W, Więski K, Krawczyk B 2006 Systematic position of copepods of the genus *Achtheres* (Crustacea: Copepoda: Siphonostomatoida) parasitizing perch, *Perca fluviatilis* L., and zander, *Sander lucioperca* (L.). J. Fish Dis. 29, 103–113. (10.1111/j.1365-2761.2006.00693.x) 16436121

[66] Blanco-Bercial L, Álvarez-Marqués F 2007 RFLP procedure to discriminate between *Clausocalanus* Giesbrecht, 1888 (Copepoda, Calanoida) species in the Central Cantabrian Sea. J. Exp. Mar. Biol. Ecol. 344, 73–77.

[67] Smolina I, Kollias S, Poortvliet M, Nielsen TG, Lindeque P, Castellani C, Møller EF, Blanco-Bercial L, Hoarau G. 2014 Genome- and transcriptome-assisted development of nuclear insertion/deletion markers for *Calanus* species (Copepoda: Calanoida) identification. Mol. Ecol. Res. 14, 1072–1079. (10.1111/1755-0998.12241) 24612683

[68] Choquet M et al. 2017 Genetics redraws pelagic biogeography of *Calanus*. Biol. Lett. 13, 20170588 (10.1098/rsbl.2017.0588) 29263132 PMC5746537

[69] Lindeque P, Harris RP, Jones MB, Smerdon GR 2004 Distribution of *Calanus* spp. as determined using a genetic identification system. Scientia Marina 68(Suppl. 1), 121–128. (10.3989/scimar.2004.68s1121)

[70] Weydmann A, Coelho NC, Ramos AA, Serrão EA, Pearson GA 2014 Microsatellite markers for the Arctic copepod *Calanus glacialis* and cross-amplification with *C. finmarchicus*. Conserv. Genet. Resour. 6, 1003–1005. (10.1007/s12686-014-0269-6)

[71] Andrews KR, Norton EL, Fernandez-Silva I, Portner E, Goetze E 2014 Multilocus evidence for globally distributed cryptic species and distinct populations across ocean gyres in a mesopelagic copepod. Mol. Ecol. 23, 5462–5479. (10.1111/mec.12950) 25283587

[72] Hirai J 2019 Insights into reproductive isolation within the pelagic copepod *Pleuromamma abdominalis* with high genetic diversity using genome-wide SNP data. Mar. Biol. 167, 1 (10.1007/s00227-019-3618-x)

[73] Hebert PDN, Ratnasingham S, de Waard RJ 2003 Barcoding animal life: cytochrome *c* oxidase subunit 1 divergences among closely related species. Proc. R. Soc. Lond. B (Suppl.) 270, S96–S99. (10.1098/rsbl.2003.0025) PMC1698023 12952648

[74] Hebert PDN, Cywinska A, Ball SL, DeWaard JR 2003 Biological identifications through DNA barcodes. Proc. R. Soc. B 270, 313–321. (10.1098/rspb.2002.2218) PMC1691236 12614582

[75] Bucklin A, Frost WB, Kocher TD 1992 DNA sequence variation of the mitochondrial 16S rRNA in *Calanus* (Copepoda; Calanoida): intraspecific and interspecific patterns. Mol. Mar. Biol. Biotech. 1, 397–407.

[76] Bucklin A, LaJeunesse TC 1994 Molecular genetic variation of *Calanus pacificus* (Copepoda: Calanoida): preliminary evaluation of genetic structure and subspecific differentiation based on mtDNA sequences. CalCOFI Rep. 35, 45–51.

[77] Bucklin A, Frost BW, Kocher TD 1995 Molecular systematics of six *Calanus* and three *Metridia* species (Calanoida: Copepoda). Mar. Biol. 121, 655–664. (10.1007/BF00349301)

[78] Folmer O, Black M, Hoeh W, Lutz R, Vrijenhoek R 1994 DNA primers for amplification of mitochondrial cytochrome *c* oxidase subunit I from diverse metazoan invertebrates. Mol. Mar. Biol. Biotech. 3, 294–299. 7881515

[79] Bucklin A, Steinke D, Blanco-Bercial L 2011 DNA barcoding of marine metazoa. Annu. Rev. Mar. Sci. 3, 471–508. (10.1146/annurev-marine-120308-080950) 21329214

[80] Bucklin A, Ortman BD, Jennings RM, Nigro LM, Sweetman CJ, Copley NJ, Sutton T, Wiebe PH 2010 A “Rosetta Stone” for metazoan zooplankton: DNA barcode analysis of species diversity in the Sargasso Sea (Northwest Atlantic Ocean). Deep-Sea Res. Part II 57, 2234–2247. (10.1016/j.dsr2.2010.09.025)

[81] Bucklin A, Hopcroft RR, Kosobokova KN, Nigro LM, Ortman BD, Jennings RM, Sweetman CJ 2010 DNA barcoding of Arctic Ocean holozooplankton for species identification and recognition. Deep Sea Res. Part II 57, 40–48. (10.1016/j.dsr2.2009.08.005)

[82] DeSalle R, Egan MG, Siddall M 2005 The Unholy Trinity: taxonomy, species delimitation and DNA barcoding. Phil. Trans. R. Soc. B 360, 1905–1916. (10.1098/rstb.2005.1722) 16214748 PMC1609226

[83] Goetze E, Bradford-Grieve J 2005 Genetic and morphological description of *Eucalanus spinifer* T. Scott, 1894 (Calanoida: Eucalanidae), a circumglobal sister species of the copepod *E. hyalinus* s.s. (Claus, 1866). Prog. Oceanogr. 65, 55–87. (10.1016/j.pocean.2005.02.015)

[84] Goetze E, Ohman MD 2010 Integrated molecular and morphological biogeography of the calanoid copepod family Eucalanidae. Deep Sea Res. Part II. 57, 2110–2129. (10.1016/j.dsr2.2010.09.014)

[85] Stupnikova AN, Molodtsova TN, Mugue NS, Neretina TV 2013 Genetic variability of the *Metridia lucens* complex (Copepoda) in the Southern Ocean. J. Mar. Sys. 128, 175–184. (10.1016/j.jmarsys.2013.04.016)

[86] Ueda H, Bucklin A 2006 *Acartia (Odontacartia) ohtsukai*, a new brackish-water calanoid copepod from Ariake Bay, Japan, with a redescription of the closely related *A. pacifica* from the Seto Inland Sea. Hydrobiologia 560, 77–91. (10.1007/s10750-005-9513-0)

[87] Eyun S-i, Lee Y-H, Suh H-L, Kim S, Soh HY 2007 Genetic identification and molecular phylogeny of *Pseudodiaptomus* species (Calanoida, Pseudodiaptomidae) in Korean Waters. Zool. Sci. 24, 265–271. (10.2108/zsj.24.265) 17551247

[88] Lindeque PK, Hay SJ, Heath MR, Ingvarsdottir A, Rasmussen J, Smerdon GR, Waniek JJ 2006 Integrating conventional microscopy and molecular analysis to analyse the abundance and distribution of four *Calanus* congeners in the North Atlantic. J. Plankton Res. 28, 221–238. (10.1093/plankt/fbi115)

[89] Bradford-Grieve JM, Blanco-Bercial L, Boxshall GA 2017 Revision of Family Megacalanidae (Copepoda: Calanoida). Zootaxa 4229, 183 (10.11646/zootaxa.4229.1.1) 28187560

[90] Kozol R, Blanco-Bercial L, Bucklin A 2012 Multi-Gene analysis reveals a lack of genetic divergence between *Calanus agulhensis* and *C. sinicus* (Copepoda; Calanoida). PLoS ONE 7, e45710 (10.1371/journal.pone.0045710) 23118849 PMC3485259

[91] Viñas MD, Blanco-Bercial L, Bucklin A, Verheye H, Bersano JGF, Ceballos S 2015 Phylogeography of the copepod *Calanoides carinatus* s.l. (Krøyer) reveals cryptic species and delimits *C. carinatus* s.s. distribution in SW Atlantic Ocean. J. Exp. Mar. Biol. Ecol. 468, 97–104. (10.1016/j.jembe.2015.03.012)

[92] Bradford-Grieve JM, Blanco-Bercial L, Prusova I 2017 *Calanoides natalis* Brady, 1914 (Copepoda: Calanoida: Calanidae): identity and distribution in relation to coastal oceanography of the eastern Atlantic and western Indian Oceans. J. Nat. Hist. 51, 807–836. (10.1080/00222933.2017.1296198)

[93] Böttger-Schnack R, Machida RJ 2011 Comparison of morphological and molecular traits for species identification and taxonomic grouping of oncaeid copepods. Hydrobiologia 666, 111–125. (10.1007/s10750-010-0094-1)

[94] Goetze E 2010 Species discovery in marine planktonic invertebrates through global molecular screening. Mol. Ecol. 19, 952–967. (10.1111/j.1365-294X.2009.04520.x) 20089123

[95] Fonseca VG et al. 2010 Second-generation environmental sequencing unmasks marine metazoan biodiversity. Nat. Commun. 1, 1–8. (10.1038/ncomms95) 20981026 PMC2963828

[96] Mohrbeck I, Raupach MJ, Martínez Arbizu P, Knebelsberger T, Laakmann S 2015 High-throughput sequencing—the key to rapid biodiversity assessment of marine metazoa? PLoS ONE 10, e0140342 (10.1371/journal.pone.0140342) 26479071 PMC4610693

[97] Sommer SA, Van Woudenberg L, Lenz PH, Cepeda G, Goetze E 2017 Vertical gradients in species richness and community composition across the twilight zone in the North Pacific Subtropical Gyre. Mol. Ecol. 26, 6136–6156. (10.1111/mec.14286) 28792641

[98] Brown EA, Chain FJ, Crease TJ, MacIsaac HJ, Cristescu ME 2015 Divergence thresholds and divergent biodiversity estimates: can metabarcoding reliably describe zooplankton communities? Ecol. Evol. 5, 2234–2251. (10.1002/ece3.1485) 26078859 PMC4461424

[99] Sun C, Zhao Y, Li HL, Haitao DY, MacIsaac HI, Zhan A 2015 Unreliable quantitation of species abundance based on high-throughput sequencing data of zooplankton communities. Aquatic Biol. 24, 9–15. (10.3354/ab00629)

[100] Pearman JK, El-Sherbiny MM, Lanzén A, Al-Aidaroos AM, Irigoien X 2014 Zooplankton diversity across three Red Sea reefs using pyrosequencing. Front. Marine Sci. 1, 27 (10.3389/fmars.2014.00027)

[101] Pearman JK, Irigoien X 2015 Assessment of zooplankton community composition along a depth profile in the Central Red Sea. PLoS ONE 10, e0133487 (10.1371/journal.pone.0133487) 26186220 PMC4506081

[102] Albaina A, Aguirre M, Abad D, Santos M, Estonba A 2016 18S rRNA V9 metabarcoding for diet characterization: a critical evaluation with two sympatric zooplanktivorous fish species. Ecol. Evol. 6, 1809–1824. (10.1002/ece3.1986) 27087935 PMC4801955

[103] Abad D, Albaina A, Aguirre M, Laza-Martínez A, Uriarte I, Iriarte A, Villate F, Estonba A 2016 Is metabarcoding suitable for estuarine plankton monitoring? A comparative study with microscopy. Mar. Biol. 163, 149 (10.1007/s00227-016-2920-0)

[104] Abad D, Albaina A, Aguirre M, Estonba A 2017 18S V9 metabarcoding correctly depicts plankton estuarine community drivers. Mar. Ecol. Prog. Ser. 584, 31–43. (10.3354/meps12373)

[105] Casas L, Pearman JK, Irigoien X 2017 Metabarcoding reveals seasonal and temperature-dependent succession of zooplankton communities in the Red Sea. Front. Mar. Sci. 4, 241 (10.3389/fmars.2017.00241)

[106] Hirai J, Katakura S, Kasai H, Nagai S 2017 Cryptic zooplankton diversity revealed by a metagenetic approach to monitoring metazoan communities in the coastal waters of the Okhotsk Sea, Northeastern Hokkaido. Front. Mar. Sci. 4, 379 (10.3389/fmars.2017.00379)

[107] Bucklin A, Yeh HD, Questel JM, Richardson DE, Reese B, Copley NJ, Fields D 2019 Time-series metabarcoding analysis of zooplankton diversity of the NW Atlantic continental shelf. ICES J. Mar. Sci. 76, 1162–1176. (10.1093/icesjms/fsz021)

[108] Clarke LJ, Beard JM, Swadling KM, Deagle BE 2017 Effect of marker choice and thermal cycling protocol on zooplankton DNA metabarcoding studies. Ecol. Evol. 7, 873–883. (10.1002/ece3.2667) 28168024 PMC5288259

[109] Djurhuus A, Pitz K, Sawaya NA, Rojas-Marquez J, Michaud B, Montes E, Muller-Karger F, Breitbart M 2018 Evaluation of marine zooplankton community structure through environmental DNA metabarcoding. Limnol. Oceanogr. Methods 16, 209–221. (10.1002/lom3.10237) 29937700 PMC5993268

[110] Stefanni S, Stanković D, Borme D, de Olazabal A, Juretić T, Pallavicini A, Tirelli V 2018 Multi-marker metabarcoding approach to study mesozooplankton at basin scale. Sci. Rep. 8, 12085 (10.1038/s41598-018-30157-7) 30108256 PMC6092319

[111] Berry TE et al. 2019 Marine environmental DNA biomonitoring reveals seasonal patterns in biodiversity and identifies ecosystem responses to anomalous climatic events. PLoS Genet. 15, e1007943 (10.1371/journal.pgen.1007943) 30735490 PMC6368286

[112] Carroll EL et al. 2019 Multi-locus DNA metabarcoding of zooplankton communities and scat reveal trophic interactions of a generalist predator. Sci. Rep. 9, 281 (10.1038/s41598-018-36478-x) 30670720 PMC6342929

[113] Zhang GK, Chain FJJ, Abbott CL, Cristescu ME 2018 Metabarcoding using multiplexed markers increases species detection in complex zooplankton communities. Evol. Appl. 11, 1901–1914. (10.1111/eva.12694) 30459837 PMC6231476

[114] Hirai J, Shimode S, Tsuda A 2013 Evaluation of ITS2-28S as a molecular marker for identification of calanoid copepods in the subtropical western North Pacific. J. Plankton Res. 35, 644–656. (10.1093/plankt/fbt016)

[115] Hirai J, Yasuike M, Fujiwara A, Nakamura Y, Hamaoka S, Katakura S, Takano Y, Nagai S 2015 Effects of plankton net characteristics on metagenetic community analysis of metazoan zooplankton in a coastal marine ecosystem. J. Exp. Mar. Biol. Ecol. 469, 36–43. (10.1016/j.jembe.2015.04.011)

[116] Taberlet P, Bonin A, Zinger L, Coissac E 2018 Environmental DNA: For biodiversity research and monitoring. Oxford, UK: Oxford University Press.

[117] Günther B, Knebelsberger T, Neumann H, Laakmann S, Martínez Arbizu P 2018 Metabarcoding of marine environmental DNA based on mitochondrial and nuclear genes. Sci. Rep. 8, 14822 (10.1038/s41598-018-32917-x) 30287908 PMC6172225

[118] Lacoursiere-Roussel A et al. 2018 eDNA metabarcoding as a new surveillance approach for coastal Arctic biodiversity. Ecol. Evol. 8, 7763–7777. (10.1002/ece3.4213) 30250661 PMC6144963

[119] Goldberg CS et al. 2016 Critical considerations for the application of environmental DNA methods to detect aquatic species. Methods Ecol. Evol. 7, 1299–1307. (10.1111/2041-210X.12595)

[120] Deiner K et al. 2017 Environmental DNA metabarcoding: transforming how we survey animal and plant communities. Mol. Ecol. 26, 5872–5895. (10.1111/mec.14350) 28921802

[121] Santoferrara LF 2019 Current practice in plankton metabarcoding: optimization and error management. J. Plankton Res. 41, 571–582. (10.1093/plankt/fbz041)

